# A Base for a Richmond Crown

**Published:** 1907-06-15

**Authors:** James E. Boyd

**Affiliations:** Cincinnati, Ohio


### A Base for a Richmond Crown.

BY JAMES E. BOYD D.D.S., CINCINNATI, OHIO.

This crown base was intended for porcelain, but can be used equally well for gold. First prepare the root in in the usual way, except in front, when you cut it sufficiently short to allow the gum to entirely cover the joint. Prepare the canal to receive a Logan crown pin, making it fit per fectly so that it will require some force to remove it.

Then take a piece of platinum plate (33 gauge) sufficiently large to extend over the margin of the root, to bend down to make the band, punch a hole near the center and force the pin through it and burnish until it conforms to the exposed end of the root, having the pin in position; remove them both together and solder with pure gold. Then put it back in position, and with a large plugger in the automatic drive the platinum disk down against the root until you have outlined the exact size of the root on the under side of the disk; remove, and with a pair of plate shears cut a number of slits from the periphery of the disk to the outline of the root, and with a small pair of flat-nosed pliers bend the slit edge of the disk to form the band, allowing the slits to overlap each other. Place it back in position, and with a large foot plugger in the automatic drive the partially formed band close around the root, remove again, and with a wheel on the engine grind the overlapped edge of the band to a feather edge. Then take a strip of platinum plate (36) gauge just as wide as you are making the band, and with a pair of pliers hold the center of the strip against the palatine or back portion of the feather-edge band, and solder together at that point with pure gold; then with the fingers press the ends of the strip down close around the sides of the band, holding in position while soldering. This strip and gold not only reinforce ,but restore the lost form of the root, and when properly executed make a perfect-fitting base for a crown.—*Dental Summary.*

